# The impact of convergence insufficiency on selective visual attention among university students

**DOI:** 10.1371/journal.pone.0336715

**Published:** 2025-11-25

**Authors:** Mohammed M. Alnawmasi

**Affiliations:** Department of Optometry, College of Applied Medical Sciences, Qassim University, Buraydah, Saudi Arabia; The Ohio State University, UNITED STATES OF AMERICA

## Abstract

Convergence insufficiency (CI) is a common binocular vision disorder that impairs near vision-related tasks and is associated with symptoms such as eye strain, headaches, and reading difficulties. This study examined the effect of CI on selective visual attention by comparing performance on a modified visual search task between patients with CI and visually normal controls. A total of 42 male university students participated, including 20 patients diagnosed with CI and 22 age-matched controls. Participants completed a computerized visual search task involving different levels of target-distractor similarity and number of distractors. Accuracy (percent correct) and reaction time (RT) were recorded across task conditions. Reaction time was significantly slower in the CI group across all task conditions (mean RT: 1.21 ± 0.15 s) compared to controls (mean RT: 0.97 ± 0.12 s; main effect of group: F(1,160) = 38.2, p < 0.0001), while accuracy did not significantly differ between groups (CI: 86.3% ± 5.7; controls: 87.1% ± 6.1; F(1,160) = 0.002, p = 0.97). Task difficulty significantly influenced both accuracy (F(3,160) = 37.9, p < 0.001) and reaction time (F(3,160) = 5.1, p = 0.002), but no interaction effects were observed. Moreover, linear regression revealed a significant positive correlation between CISS scores and reaction time within the CI group (R² = 0.30, p = 0.01), indicating that higher symptom severity was associated with greater cognitive processing delay. In conclusion, CI has a negative impact on processing speed but not on accuracy during attentionally demanding tasks. These findings have implications for academic performance and highlight the importance of considering visual processing speed in clinical assessments and interventions for CI.

## Introduction

Convergence insufficiency (CI) is a common binocular vision disorder, with an estimated prevalence of approximately 8% in the general population [1,2]. CI refers to difficulties in maintaining sufficient convergence for comfortable near-binocular vision. It is characterized by exophoria larger at near than at distance, a near point of convergence NPC greater than 5 cm, and reduced positive fusional vergence BFV at near [3]. These clinical signs are often accompanied by vision-related symptoms such as double vision, blur, headaches, and visual fatigue, which are typically quantified using the Convergence Insufficiency Symptom Survey (CISS) [4,5]. Because these symptoms primarily occur during sustained near tasks, CI is known to disrupt daily activities such as reading, computer use, and other forms of prolonged near work [6,7].

The functional consequences of CI can extend to academic performance. For example, Kovarski et al (2020) investigated the effect of biocular visual disorders on academic achievement among teenagers. They showed a link between binocular vision-related symptoms and academic achievement in which those with higher symptom scores had poor academic performance compared to those with lower symptom scores. On the other hand, clinical trials showed a significant reduction in symptoms following successful treatment of CI where the improvement of CI-related symptoms was associated with improved academic performance [8,9]. However, previous studies have not investigated whether improved academic performance was related to improvement in specific cognitive domains rather than overall improvement in academic performance.

Academic achievement is known to be influenced by different factors, including cognitive skills such as visual attention [10]. The term “visual attention” refers to a group of cognitive mechanisms that limit the processing of visual information to selected visual stimuli [11]. The latter appears to have significant effects on several domains important to academic foundations, including language, literacy, and mathematics.

Neuroimaging studies have shown that CI is associated with reduced activation in multiple brain regions, including the cuneus, secondary visual cortex (V2), parietal eye fields (PEF), posterior parietal cortex, and frontal eye fields (FEF), during vergence tasks [12,13]. These areas are not only integral to oculomotor control but also play a key role in attentional networks, responsible for allocating visual resources to relevant stimuli and suppressing irrelevant input [14,15]. Thus, dysfunction in these regions may result in attentional deficits as well as impaired fusional vergence dynamics during tasks requiring sustained near work, such as reading or studying. For example, slower vergence peak velocities have been correlated with reduced functional activity in the frontal eye fields, posterior parietal cortex, and cerebellar vermis which are key regions supporting both oculomotor control and attentional processes, suggesting that CI-related dysfunction in these areas may impair the neural mechanisms of selective attention [16].

Therapeutic interventions also provide insight into the link between CI and selective visual attention. For example, office-based vergence and accommodative therapy (OBVAT) has been shown to improve clinical measures such as near point of convergence and fusional vergence, and ongoing randomized trials (e.g., CITT-ART) are evaluating whether these improvements translate into better attention and reading performance [9]. While the precise neural mechanisms remain to be clarified, prior evidence suggests that vision therapy interventions for CI can reduce inattention and ADHD-like behaviors in children, supporting a potential role for vision therapy in strengthening attentional control [17]. These improvements suggest that vision therapy may restore not just oculomotor function but also attentional control by re-engaging visual and frontoparietal networks. Similarly, virtual reality (VR)-based therapy has been shown to improve convergence and reduce symptoms, with the added advantage of actively engaging attentional systems through immersive, interactive environments [18]. This reinforces the idea that CI treatment exerts a dual effect on motor alignment and selective attention processing. Moreover, CI is more prevalent in neurological disorders such as traumatic brain injury (TBI), Parkinson’s disease (PD), autism spectrum disorder (ASD), and in older adults with cognitive impairment which supports that CI arises from impaired function in these subcortical and cortical areas [19–22].

There are limited studies that directly assess selective visual attention in patients with CI. One notable investigation by Daniel and Kapoula (2016) demonstrated that individuals with CI exhibited significantly greater Stroop interference effects compared to those with normal binocular vision [23]. The Stroop test, a widely used measure of executive function and attention, requires participants to inhibit the automatic response to read a word in order to correctly name the ink color in which it is printed. This increased interference effect in CI highlights the vulnerability of attentional control when binocular vision mechanisms are compromised. Importantly, they found a strong correlation between positive fusional vergence (PFV) and interference performance, suggesting that reduced vergence capacity exacerbates difficulties in inhibitory control. Because both vergence and executive control rely on overlapping parietofrontal cortical areas, this work provides evidence that vergence dysfunctions extend beyond visual alignment problems and compromise cognitive executive functions, including attentional control. In a different study, participants with higher Convergence Insufficiency Symptom Survey (CISS) scores showed slower reading speed and elevated Stroop interference effects [24]. These findings strengthen the rationale for examining selective visual attention in CI populations, as disrupted vergence may impair the neural mechanisms that underlie both efficient reading and cognitive control. Therefore, this study aimed to assess visual attention using a visual search task with a different level of distractions in a group of patients with CI without neurological disorders and normal-sighted controls. This study hypothesized that patients with CI would be less accurate and respond slower while performing selective visual attention tasks compared to controls.

## Method

### Participants

Based on the calculated effect size (Cohen’s d) derived from the Daniel & Kapoula (2016) study that reported significant deficits in selective visual attention among individuals with binocular vision disorders, the observed effect was large (d = 0.94) [23]. Accordingly, a sample size of 42 participants provides sufficient statistical power when assessed using a selective visual attention task, under the assumption of a power level of 0.80.

The current study included 20 participants who were newly diagnosed with CI and 22 control participants. Participants were recruited from the optometry clinics at the College of Applied Medical Sciences, Qassim University between January 2025 and April 2025. Major eligibility criteria for the diagnosis of CI include the following: (a) greater exophoria at near-than distance by 4 PD; (b) receded near point of convergence (>6 cm breakpoint); (c) minimum normative positive fusional vergence at near (<15 PD for break); and CISS score of 16 or higher [3].

**Fig 1:**
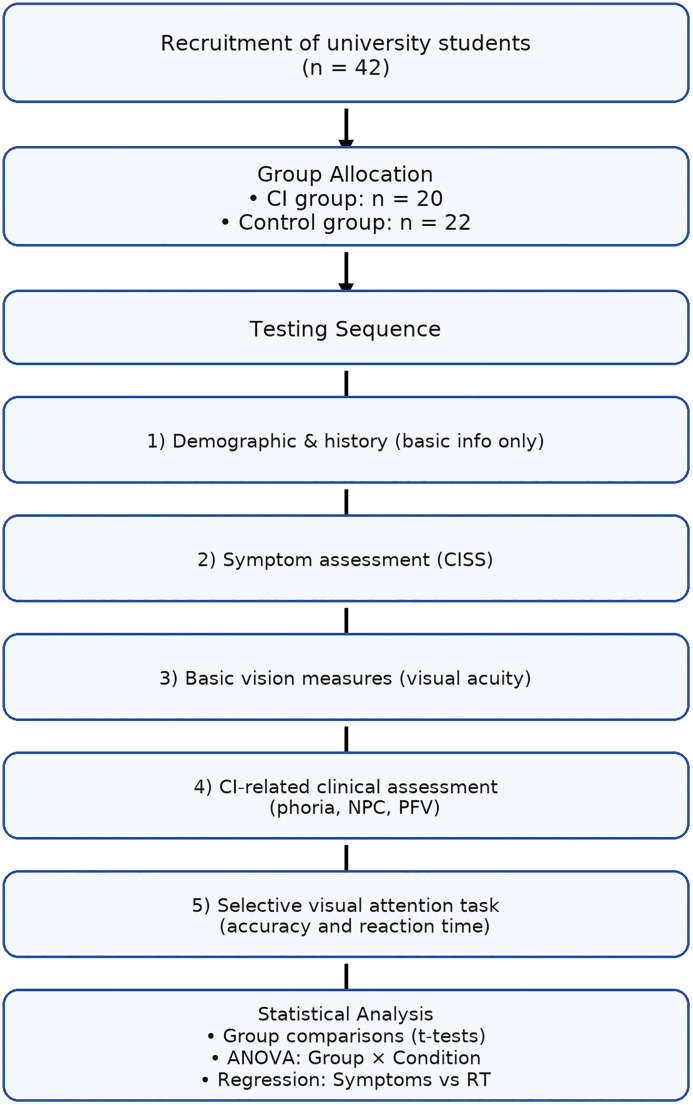
A diagram outlining the sequential stages of participant recruitment, allocation, assessment, and analysis.

Exclusion criteria were binocular visual acuity worse than 6/12, strabismus, ocular surgery, neurological disorders, Attention Deficit Hyperactivity Disorder (ADHD), learning disabilities or a diagnosis of cognitive impairment. Preliminary information, such as the general health and ocular history was gathered at the beginning of the study. This study was reviewed and received clearance through the Qassim University Research Ethics Committee (#24-08-09). All participants provided written informed consent prior to participation after receiving a full explanation of the study procedures and objectives. Fig 1 summarizes the methodology of the current study, including participant recruitment, group allocation, testing sequence, and statistical analyses.

### Selective visual attention assessment

**Fig 2:**
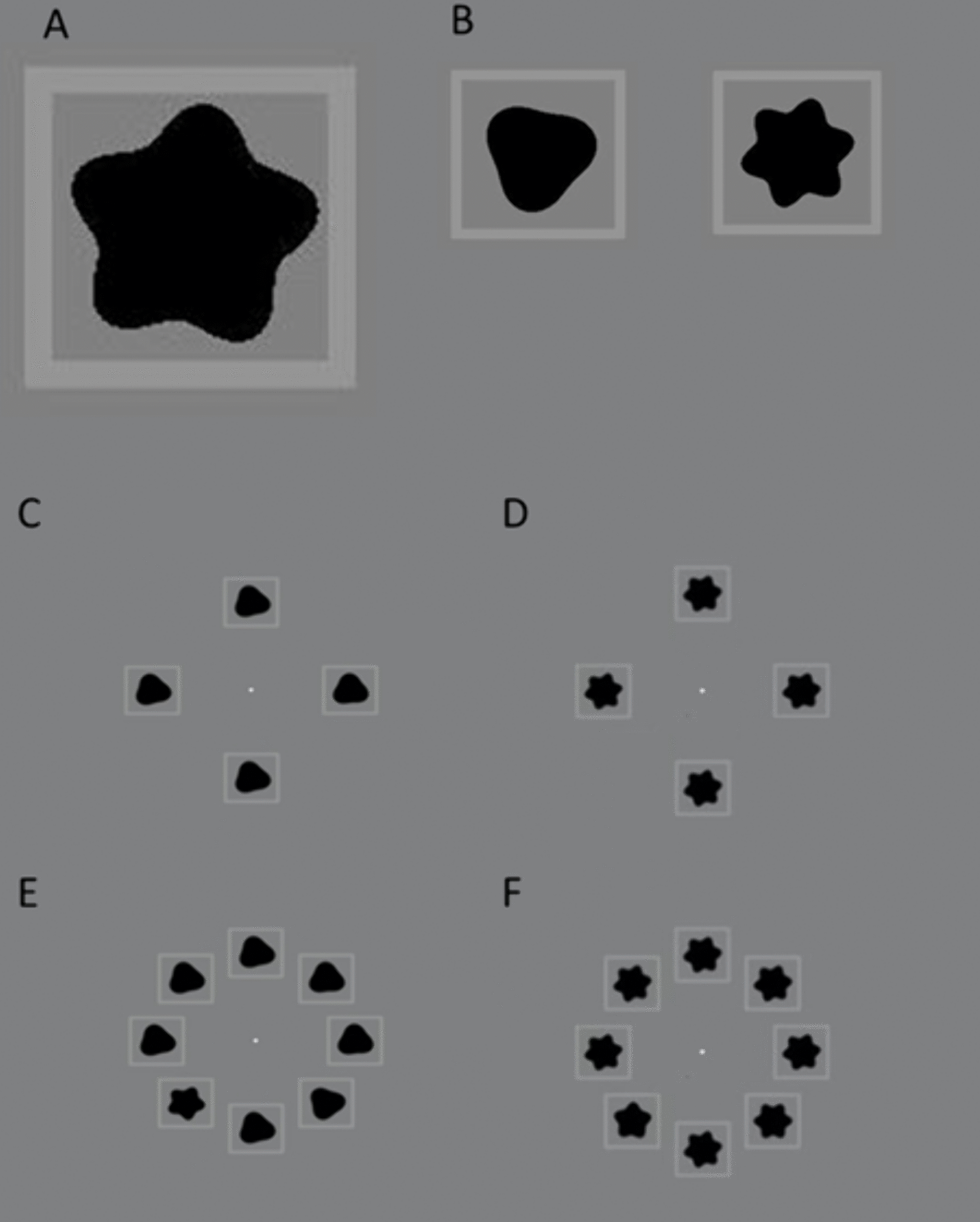
An illustration of the radial frequency RF patterns used in the search stimulus. A shows the RF stimulus presented as a target (5 RF patterns) while B shows the different RF distractors (3 and 4 RF patterns). C & 1D show the search stimulus where there are 3 distractors while E & 1F show the search stimulus where there are 7 distractors. In E the target stimulus was present while in F the target stimulus was absent.

A modified visual search task was used to assess selective visual attention. This task has been used and validated previously [25,26]. The search stimuli were generated in MATLAB (MathWorks, 2018), using the Psychophysics Toolbox extensions. In this task, the participants were asked to determine whether a specific Radial frequency RF pattern was present or absent among other patterns of RF distractors, see Fig 2. The RF pattern for the target was fixed (RF = 5) and was varied for distractors to modulate task difficulty (RF = 3 or 4). The number of distractors was also varied in which the target was embedded among either 3 or 7 distractors. The task trial was repeated for different conditions where the difficulty of the visual search task was variable.

**Fig 3:**
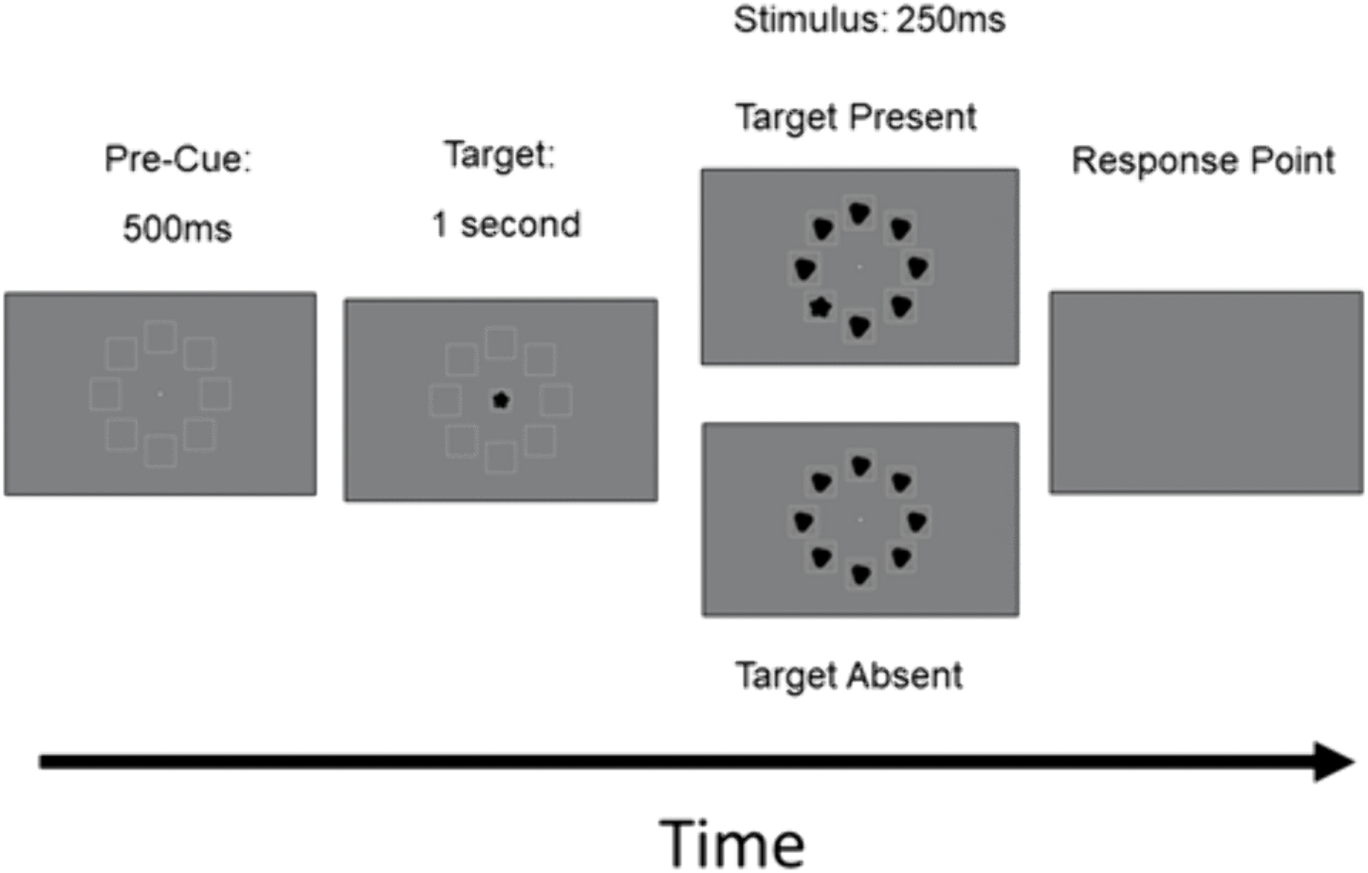
A schematic diagram for the main task conditions. The target stimulus was initially presented for 1 second, followed by the search stimulus, which was presented for 250 ms. The task of the participant was to indicate whether the target stimulus was present or absent.

A schematic of the main task conditions is provided in Fig 3. At the beginning of the trial, the target was presented centrally for one second. This was followed by the presentation of the search stimulus in which the target appeared in a different location. In absent trials, no target was presented and all shapes were distractor RF patterns. After the stimulus appeared, the screen turned grey and participants had to decide whether the target was present or absent.

There were two main outcome measures: accuracy (percentage of correct responses) and reaction time (RT, measured in seconds). These two outcome measures are typically used as performance measures of selective visual attention.

### Statistical analysis

Statistical analyses were conducted using GraphPad Prism (Graph Pad Software Inc., San Diego, CA). The normality of the data was evaluated using the Shapiro–Wilk test, and homogeneity of variances was assessed using Levene’s test; both analyses confirmed that the data met the assumptions of normal distribution and equal variances. Three analyses were conducted. Firstly, independent samples t-tests (parametric) were used to compare the two groups’ age, symptom scores, and CI-related clinical findings. Secondly, 2-way ANOVA with factors group (CI x Controls) and search conditions (1, 2, 3, 4) was used to compare the different search conditions between CI and control groups. This analysis was conducted for both outcome measures (accuracy and reaction time). Thirdly, linear regression analyses were performed to determine the relationship between performance on the visual attention task and CISS symptom scores.

## Results

**Fig 4:**
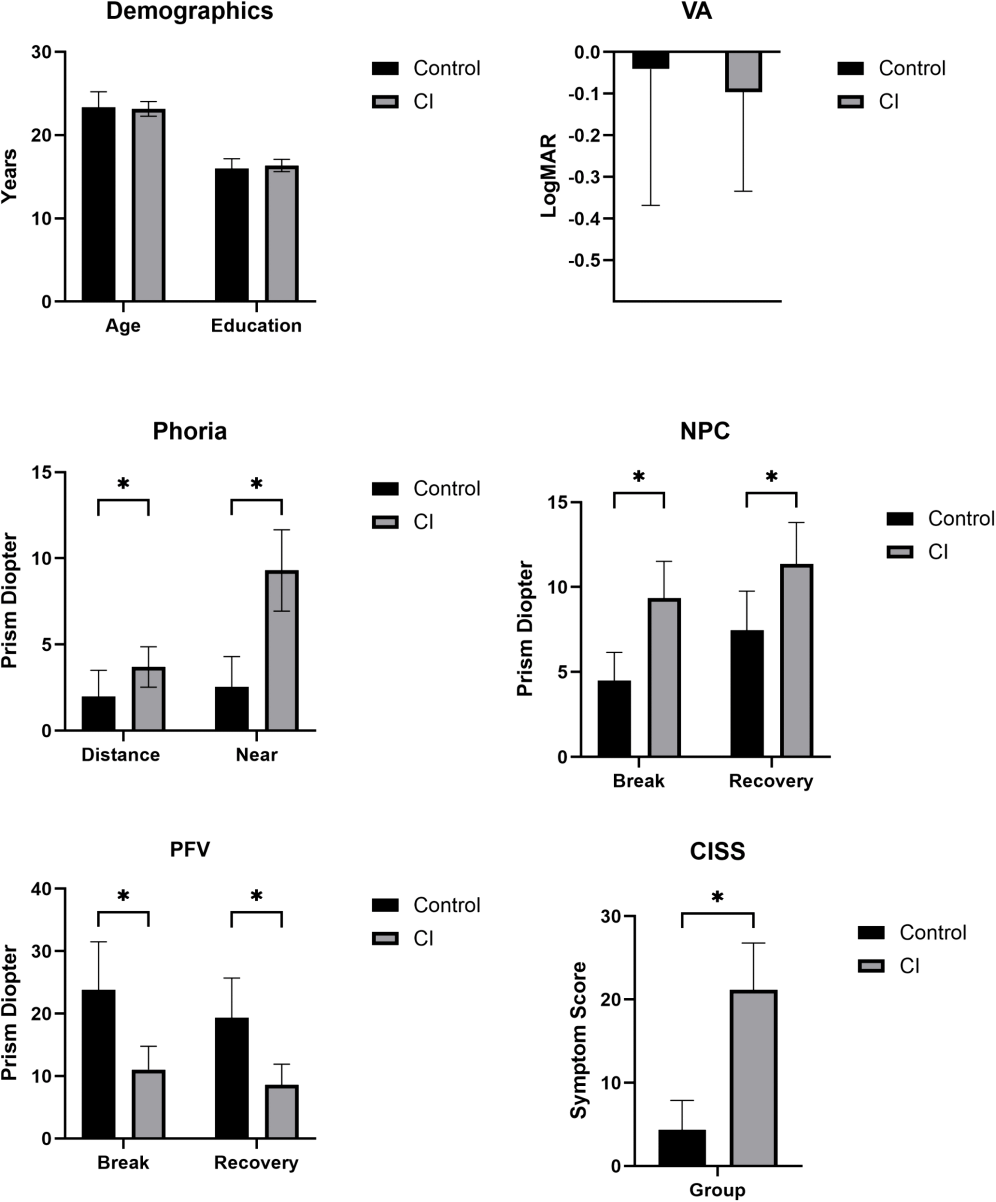
Descriptive statistics for demographics and CI-related clinical findings for groups. Bars represent group means ± standard deviation (SD), and asterisks indicate significant between-group differences (p < 0.05). Abbreviations: VA, visual acuity; NPC, near point of convergence; PFV, positive fusional vergence; CISS, Convergence Insufficiency Symptom Survey; logMAR, logarithm of the minimum angle of resolution.

Of the 42 participants in this study, 22 (53%) were controls and 20 (47%) were patients with CI. There was no statistically significant difference between the control group and the patient group in terms of age and education. All participants self-reported no neurological disorders or Attention Deficit Hyperactivity Disorder (ADHD). The CI-related measures (NPC, PFV, NFV, CISS) were significantly worse in patients with CI than in controls (P value < 0.001), see Fig 4. Although the CISS score was significantly higher in the CI group, not all the participants in the CI group were symptomatic CI.

Performance on selective visual attention tasks was analyzed separately for sensitivity and reaction time outcome measures.

### Accuracy

**Fig 5:**
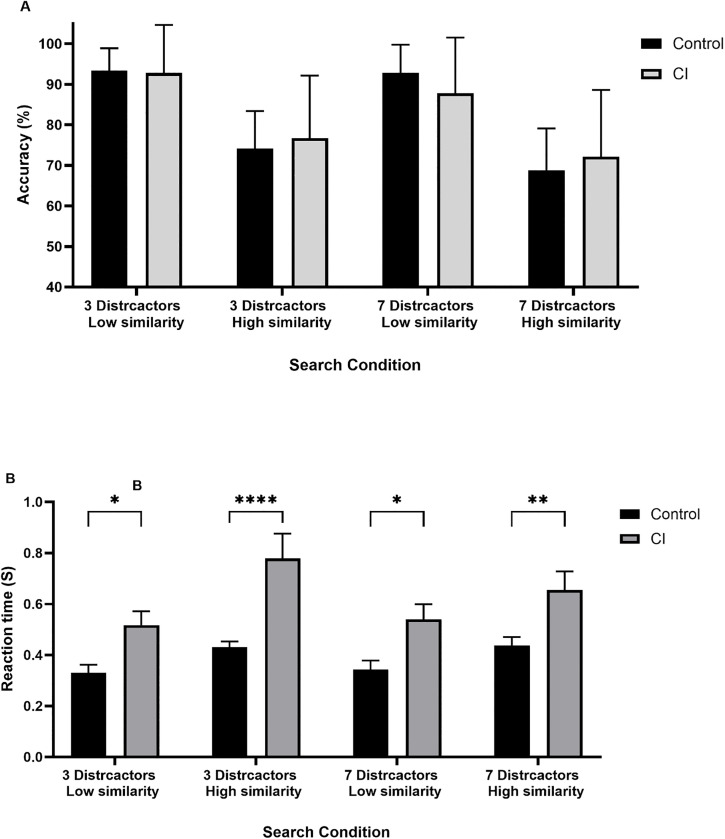
The accuracy (A) and reaction time RT (B) as a function of the search condition for control participants and patients with CI (different bars). Error bars indicate 1 standard error of the mean.

Fig 5A plots the accuracy (group average) as a function of the search condition (1, 2, 3, and 4) for patients with CI and control participants. The percentage accuracy values are indicative of task performance.

As apparent in Fig 5A, the average performance accuracy did not vary as a function of the group but varied according to the search condition for both control and CI groups. A 2-way ANOVA with the factor of Group (CI vs. control) and search condition (1,2,3,4) as factors revealed a main effect of search condition (F (3, 160) = 37.9, p < 0.001) but no significant effect of Group (F (1, 160) = 0.002, p = 0.97) nor significant interaction effect (F (3, 160) = 1.1, p = 0.33). Post-hoc analysis comparing the search condition showed that both groups performed less accurately when the target-distractor similarity increased.

### Reaction time

Fig 5B plots the RT (group average) as a function of the search condition (1, 2, 3, and 4) for patients with CI and control participants. As apparent in Fig 5B, patients with CI responded slower than control participants in all search conditions. A 2-way ANOVA with the factor of Group (CI vs. control) and search condition (1,2,3,4) as factors revealed a main effect of both search condition (F (3, 160) = 5.1, p = 0.002) and Group (F (1, 160) = 38.2, p < 0.0001) but no significant interaction effect (F (3, 160) = 0.95, p = 0.41). Post-hoc analysis comparing the search conditions showed that CI groups performed significantly slower than the control group in all search conditions.

### Correlations between the performance on the visual attention task and CISS symptom scores

**Fig 6:**
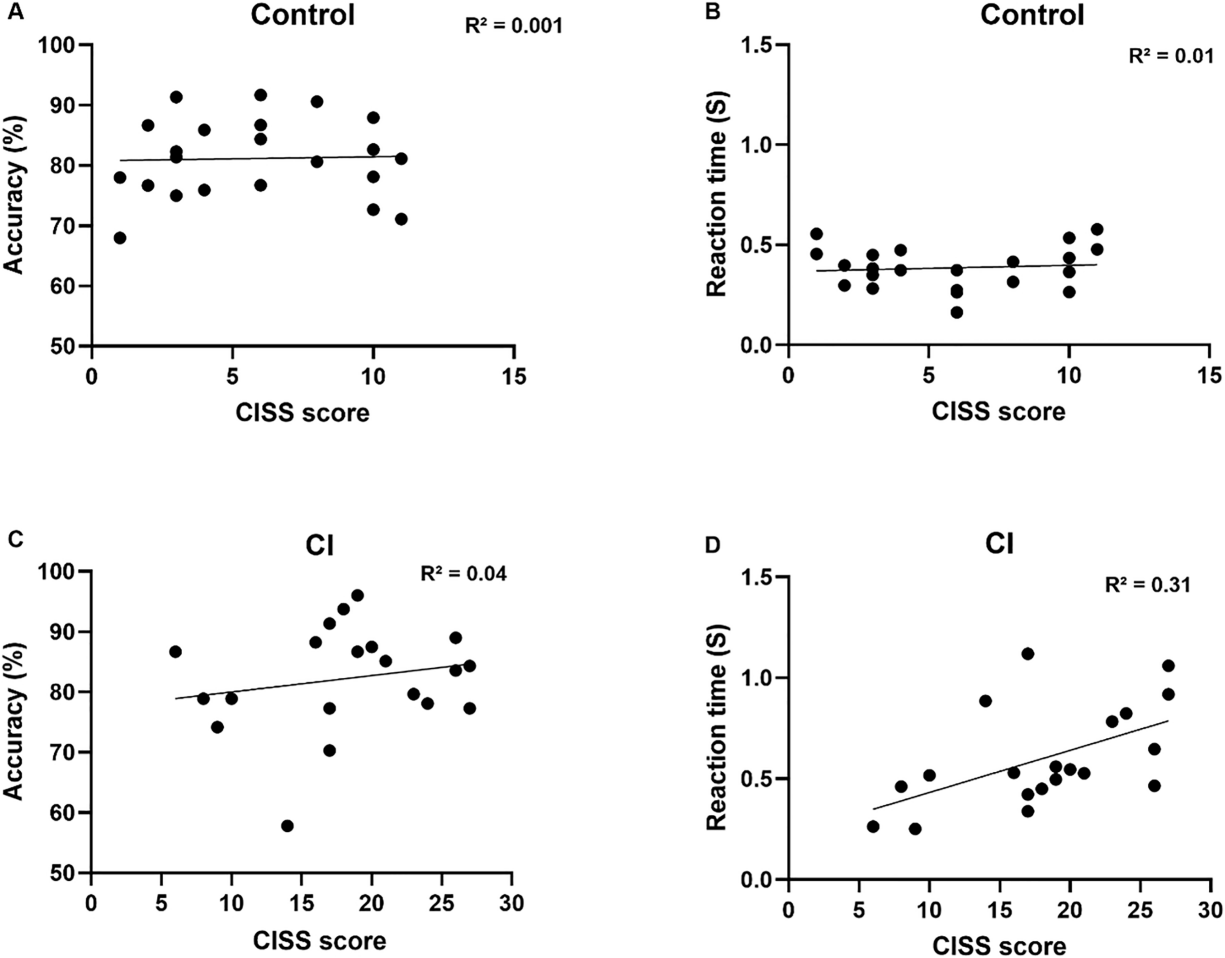
A linear regression analysis was conducted between the overall performance in the visual selective attention task (accuracy and reaction time) and the CISS score. The filled circle markers in each graph indicate individual subjects, and the solid line represents the line of best fit.

The relationship between CI-related symptoms as measured by the CISS and the performance on the visual search task among patients with CI and control participants was examined using linear regression analysis and shown in Fig 6. The reaction time while performing the visual search task was associated with the CISS score in the CI group in which patients with CI responded slower as the CI-related symptoms increased (R2 = 0.3, p = 0.01). However, in the Control group, the was no association between reaction time and the CISS score. The accuracy while performing the visual search task was also not associated with the CISS score in both groups.

## Discussion

Convergence insufficiency (CI) is a prevalent binocular vision disorder characterized by an inability to maintain proper convergence during near vision tasks. It has been linked to various vision-related symptoms that significantly impact daily activities, particularly those requiring near fixation, such as reading and computer usage. In this study, we examined the impact of CI on selective visual attention by comparing performance on a visual search task between CI patients and controls. The results revealed significant differences in reaction time but no differences in accuracy between the two groups. These findings provide insights into the cognitive mechanisms underlying CI and its effects on visual processing.

The study findings demonstrate that patients with CI exhibited significantly slower reaction times compared to controls across all search conditions, whereas accuracy did not differ between groups. This suggests that individuals with CI do not necessarily suffer from impaired visual attention in terms of accuracy but may experience delays in processing visual stimuli. The lack of significant accuracy differences indicates that CI does not essentially impair the ability to distinguish targets from distractors. However, the slower reaction times observed in CI patients suggest that they require more time to process and respond to visual stimuli. This aligns with previous research linking CI to increased visual fatigue, which can slow cognitive processing speed [27]. Moreover, the increase in reaction time correlating with higher Convergence Insufficiency Symptom Survey (CISS) scores suggests that the severity of symptoms exacerbates the delay in visual processing.

The observed reaction time differences can be linked to the neural mechanisms involved in binocular vision and selective visual attention. During near vision tasks, the coordination of efferent and afferent visual pathways, including the subcortical structures (such as the basal ganglia and thalamus) and cortical areas (such as the posterior parietal cortex and frontal eye fields), is essential for maintaining single vision [12]. Dysfunction in these areas due to CI may result in delays in processing visual stimuli, as observed in the current study. Moreover, CI has been associated with various neurological disorders, such as traumatic brain injury, Parkinson’s disease, and autism spectrum disorder [19,20,22]. These conditions often involve deficits in attention and executive function, further supporting the idea that CI may contribute to delays in selective visual attention processing. The slower reaction times observed in the CI group may thus be a consequence of inefficient neural processing in the visual and attention networks.

The results of this study have important clinical implications for the diagnosis and management of CI. Given that patients with CI exhibit prolonged reaction times in visual attention tasks, clinicians may consider incorporating visual processing speed assessments into routine evaluations. Traditional clinical tests for CI primarily focus on measuring phoria, fusional vergence, and symptom severity, but assessing reaction time may provide additional insight into the functional impact of the disorder. Furthermore, treatment interventions such as vision therapy, prism correction, and accommodative training have been shown to improve CI symptoms [8,28]. Neuroimaging studies also provide further evidence of cortical reorganization following targeted vision therapy in individuals with CI. Barberán-Bernardos et al. (2024) demonstrated that vision therapy can lead to increased functional activation and enhanced connectivity within key regions such as the frontal eye fields, posterior parietal cortex, and cerebellum, areas that play crucial roles in both oculomotor control and attentional regulation [29]. These findings suggest that therapeutic improvements in vergence function are not limited to ocular motor recovery but also reflect broader neuroplastic changes within shared oculomotor attentional networks. Consequently, the slower reaction times observed in individuals with CI may arise from reduced neural efficiency within these overlapping systems, while successful treatment could restore both CI and attentional performance through the re-engagement of these cortical pathways. Given the study findings, future research may wish to investigate whether such treatments can also enhance selective visual attention performance. If successful, this could provide further justification for including CI treatment in educational and occupational settings where visual attention plays a critical role.

### Limitations

Despite the significant findings of this study, some limitations should be addressed in future research. Firstly, the sample size was relatively small and was only restricted to male participants, which may limit the generalizability of the results. Larger-scale studies are needed to confirm the study findings and explore potential variations in different populations, such as gender, younger children, and older adults. Secondly, the current study did not assess the long-term effects of CI on visual attention performance. Longitudinal studies tracking changes in reaction time and accuracy over time, particularly following CI treatment, may provide valuable insights into CI progression and treatment efficacy. Thirdly, the current study focused exclusively on selective visual attention; other cognitive domains, such as working memory and executive function, which may also be influenced by convergence insufficiency, were not assessed, and future research may wish to consider assessing these cognitive domains. Lastly, factors such as baseline cognitive ability, academic workload, and sleep quality, which are known to affect attention and reaction time, were not directly assessed. Although participants were drawn from the same academic environment, future research may wish to include standardized measures of these variables to better isolate the specific contribution of convergence insufficiency to attentional performance.

## Conclusion

The current study provides evidence that CI affects selective visual attention by increasing reaction times without compromising accuracy. These findings highlight the importance of assessing visual processing speed in CI patients and suggest that symptom severity influences the extent of cognitive delays. Given the reported impact of CI on academic and occupational performance, future research may explore interventions aimed at improving visual function and cognitive efficiency.

## Supporting information

S1 Table Descriptive statistics for demographics and CI-related clinical findings for groups. (DOCX)

S2 Row Data (XLSX)
