# Supplementary material for: The impact of convergence insufficiency on selective visual attention among university students
Source: PLoS One. 2025 Nov 25;20(11):e0336715. doi: 10.1371/journal.pone.0336715 (PMC12646433; doi:10.1371/journal.pone.0336715)
Supplement: S1 Table — (DOCX) [file pone.0336715.s001.docx]

**S1 Table 1:** **Descriptive statistics for demographics and CI-related clinical findings for groups.**

| **Characteristic** | **Control** | **CI** | **P value** |
| --- | --- | --- | --- |
| Age (years) | 23.4 (1.8) | 23.1 (1.8) | p = 0.64 |
| Education (years) | 16.1 (1.6) | 16.3 (0.8) | p = 0.27 |
| Binocular distance visual acuity in the (logMAR) | -0.04 (0.32) | -0.09 (0.23) | p = 0.38 |
| Distance phoria (Δ) | 2 (1.5) | 3.7 (1.2) | p < 0.001 |
| Near phoria (Δ) | 2.6 (1.7) | 9.3 (2.3) | p < 0.001 |
| NPC break (cm) | 4.5 (1.6) | 9.4 (2.1) | p < 0.001 |
| NPC recovery (cm) | 7.5 (2.2) | 11.3 (2.2) | p < 0.001 |
| PFV break (Δ) | 23.9 (7.5) | 11.1 (3.7) | p < 0.001 |
| PFV recovery (Δ) | 19.3 (6.2) | 8.6 (3.2) | p < 0.001 |
| CISS Score | 4.3 (3.4) | 21.2 (5.1) | p < 0.001 |
